# Residential Water Meters as Edge Computing Nodes: Disaggregating End Uses and Creating Actionable Information at the Edge

**DOI:** 10.3390/s21165310

**Published:** 2021-08-06

**Authors:** Nour A. Attallah, Jeffery S. Horsburgh, Arle S. Beckwith, Robb J. Tracy

**Affiliations:** 1 Department of Civil and Environmental Engineering, Utah State University, 4110 Old Main Hill, Logan, UT 84322-4110, USA; jeff.horsburgh@usu.edu; 2 Utah Water Research Laboratory, Utah State University, 8200 Old Main Hill, Logan, UT 84322-8200, USA; arlebeckwith@gmail.com (A.S.B.J.); joshuatracy64@gmail.com (R.J.T.)

**Keywords:** edge computing, smart metering, residential water use

## Abstract

We present a new, open source, computationally capable datalogger for collecting and analyzing high temporal resolution residential water use data. Using this device, execution of water end use disaggregation algorithms or other data analytics can be performed directly on existing, analog residential water meters without disrupting their operation, effectively transforming existing water meters into smart, edge computing devices. Computation of water use summaries and classified water end use events directly on the meter minimizes data transmission requirements, reduces requirements for centralized data storage and processing, and reduces latency between data collection and generation of decision-relevant information. The datalogger couples an Arduino microcontroller board for data acquisition with a Raspberry Pi computer that serves as a computational resource. The computational node was developed and calibrated at the Utah Water Research Laboratory (UWRL) and was deployed for testing on the water meter for a single-family residential home in Providence City, UT, USA. Results from field deployments are presented to demonstrate the data collection accuracy, computational functionality, power requirements, communication capabilities, and applicability of the system. The computational node’s hardware design and software are open source, available for potential reuse, and can be adapted to specific research needs.

## 1. Introduction

Commercial “smart” or “intelligent” metering systems promise remote recording of water use at high temporal resolution with the potential for creating decision-relevant information (e.g., autonomously created reports and summary data products) for both water providers and consumers. However, commercially available smart meters have not yet been widely adopted in the U.S. for several reasons. First, replacing existing, analog meters with smart meters is expensive, labor-intensive, and disruptive. Second, smart meters produce “Big Data” with high volume and velocity [1]. Extracting decision-relevant information from the large volume of data produced by smart meters involves several unsolved challenges, including a critical shortage of professionals capable of working with “Big Data” to extract full value from smart metering systems. This is an instance where “Big Data” needs to be “shrunk” (i.e., summarized and mined) into information relevant to both water suppliers and consumers. The cyberinfrastructure, algorithms, and technologies to do this have not yet been well developed. In fact, many commercial smart meters collect data with high frequency but are not really “smart” in that they do not yet have the supporting cyberinfrastructure needed for fully utilizing the high frequency data they produce. Furthermore, some commercial smart metering systems limit data recording frequency to hourly intervals to reduce data volume and avoid data storage and communication bandwidth challenges. Hourly intervals are not frequent enough to identify individual water end uses (e.g., showers, toilets, faucets, etc.), limiting the utility of the data for use in understanding water use behavior and targeting efficiency measures.

Previous applications of smart metering data and associated research studies have necessarily focused on the small number of cases where cities have upgraded to newer electronic meters or where individual dataloggers can be deployed to existing meters to collect high temporal resolution data. Most of these have been conducted in Australia [2,3,4,5,6] and the United States [7,8,9,10,11,12,13,14,15]. In these studies, high-resolution water use data were collected in the field and then transferred to a centralized location for post-processing to examine residential water use behavior.

In the aforementioned non-intrusive smart metering studies [2,3,4,5,6,7,8,9,10,11,12,13,14,15], several water end use disaggregation and classification algorithms were adopted to break down the total water use registered on the household’s main meter into different water end use categories. Regardless of the algorithm adopted, the primary focus of all of these studies was to better understand the water use behavior of residential users. For example, using disaggregated and classified water end use events, references [2,6] developed a theoretical integrated water use model for understanding household water consumption, references [3,5,12,13,14] evaluated the effectiveness of implemented water demand management and rebate programs, reference [4] reconciled differences between perceived and actual residential end use water consumption, reference [7] presented an open source, low cost monitoring system for the collection of high-resolution water data on residential water meters, reference [8] estimated the probability of water fixtures being used during peak hours, references [9,15] provided a detailed understanding of how water is being used inside residential settings, and reference [10] estimated the price elasticity of water demand with water end use data.

Transferring data produced by smart water meters to a centralized location for post-processing, particularly for meters or dataloggers collecting data at a high enough temporal resolution to support end use disaggregation, has three significant limitations: (1) available bandwidth of conventional telemetry systems may be inadequate for transferring the large volume of data produced to a centralized location for post-processing, requiring technicians to visit sites to manually download data, (2) the water providing utility may not have sophisticated information technology infrastructure available to them to enable data post-processing, and (3) the utility may also lack dedicated staff and technical expertise needed to employ end use disaggregation algorithms or other sophisticated analyses.

A potential alternative to centralized information systems is to use a distributed approach, where data processing is performed at or near where the data are collected to extract and transmit only actionable data products to a centralized location. This distributed, or edge, computing approach is aimed at reducing the data management and computational burden associated with tasks such as water end-use disaggregation. By mining and summarizing the Big Data produced by smart meters at the site of data collection, required transmission bandwidth can be minimized, and derived data products can be more readily created and used to inform and improve water system management [16].

Edge computing is a distributed computing paradigm focused on bringing computing as close to the source of data as possible. Edge computing promises a range of benefits for smart Internet of Things (IoT) applications and use cases across a variety of industries. Some of the most obvious benefits of edge computing include its ability to increase network performance by processing data closer to where it is collected and reducing or eliminating the physical distance over which data must travel. Because computational tasks are performed close to where data are collected, results can be transferred to other devices with less computing power (e.g., a smart, in-home display or a centralized database system used by a public utility). This may reduce latency—defined here as the time between when the data are collected and when actionable information extracted from the data is available for use. It may also reduce the need for centralized computational resources for processing data [17].

From a security perspective, the rapid spread of edge computing devices increases the overall attack surface for networks. However, because edge computing distributes data processing and storage across a wide range of devices, it is difficult for any disruption to take down the entire network, which can provide a more secure and reliable architecture for many use cases. In addition to the speed and security advantages, edge computing offers additional advantages through scalability. Because computation is done at the edge of the network, adding additional edge devices expands computing capability without imposing large data storage and computational burdens on a centralized infrastructure and without drastically increasing network bandwidth requirements for data transfer. Additionally, since processed data products arrive at a centralized location in a usable format, requirements on water utility staff are minimized [18].

In the context of edge computing, sensor motes, or nodes, are small, affordable, low-power computer boards or microcontrollers with a radio for wireless communication [19]. Sensor motes are capable of collecting data, processing it, packaging it, and transferring it to a remote location [20]. In the current market, there are many commercially available sensor mote platforms, with some of them providing significant computational capabilities. However, most available platforms are general purpose and require significant work to adapt them for specific applications (i.e., collecting and processing data from a residential water meter).

For educational, research, and prototyping work, the Raspberry Pi platform [21], which consists of a Linux-based, single-board computer, has proven to be low cost (~$35), reliable, and adaptable for many different applications. Several groups have investigated the computational capabilities of the Raspberry Pi. For example, reference [22] developed an implementation of Multi-label classification and Random Kitchen Sink data mining algorithms on a Raspberry Pi computer using Mathematica. Implementation of Random Kitchen Sink algorithm on the Raspberry Pi computer using Mathematica improved the accuracy of Multi-label classification, reduced the code required for implementing data mining algorithms, and improved the memory usage when using large datasets. Reference [23] developed a 64-node computational cluster using a Raspberry Pi computer. Compared to conventional data-center based clusters, the computational cluster developed in [23] is low cost and low-power, portable due to its small size and weight, and has its own ambient cooling system. However, the Raspberry Pi computer’s usefulness as an edge computing device is not only because of its computational capabilities but also its ability to interface with a variety of sensors for data collection and communication peripherals for transmitting data. With available off-the-shelf electronic components, it is now much easier to design and prototype low cost and low-power devices capable of data collection, computation, and communication tasks.

In this paper, we describe the design and testing of an open source datalogger and computational node that uses the data collection and computational capabilities of modern, single-board microcontrollers and computers to turn existing, analog, and residential water meters (the vast majority of meters in use today) into battery-powered, edge computational nodes that not only collect and store high-frequency flow data but also execute algorithms for disaggregating metered flow into individual water end uses (e.g., summary totals for toilets, showers, clothes washer, etc.). The computational node is also capable of transmitting raw data and/or actionable data products to a centralized location for further analysis, interpretation, and use.

We developed the computational node as part of a larger effort aimed at developing Cyberinfrastructure for Intelligent Water Supply (CIWS). The design of the computational node builds upon our earlier work in building a simpler datalogger capable of recording data at a user-configurable temporal resolution as high as every 1 s (CIWS datalogger, [7]). Using the CIWS datalogger, data must be manually downloaded in the field and then post processed to generate useful information. For example, water end use events can be extracted from the raw trace data and classified into water end use categories using algorithms such as the CIWS disaggregator algorithm developed by [24]. To the data logging capabilities of the CIWS datalogger, the CIWS computational node described here adds the ability to run code designed to post process the data locally on the node as well as the capability to transmit raw data and/or processed data products over the Internet to a remote server. This required: (1) substantial new work on an entirely new hardware design that couples an Arduino-based data collection device with a Raspberry Pi computer to enable both data collection and edge computing capabilities; (2) addition of communication capabilities to enable transmission/telemetry of collected and/or processed data; (3) an innovative power control circuit design to enable low-power operation of tandem data collection and computational/communication devices; (4) an entirely new printed circuit board (PCB) design for manufacturing the computational node device; (5) an entirely new software design for the computational component that enables data recording, execution of arbitrary data processing code, and transmission of recorded data and/or processed results; and (6) a new case study using a water end use disaggregation and classification algorithm executed on the node and field test the demonstrates successful deployment to the field. In our case study application, we demonstrate how water end uses can be identified and classified by the node, but we designed the data processing capabilities of the computational node to be generic and support any data processing code that may be needed.

In the following sections, we describe the CIWS computational node, its operating principles, hardware design, software, and user interface (Section 2). In Section 3, we describe the methods we used for testing our prototypes within a laboratory setting using multiple water meters from different manufacturers. We then describe the results of a field deployment campaign used to assess the capabilities of the computational node under typical field operating conditions (Section 4). The final section presents discussion and conclusions. The Data Availability section provides a link where readers can find: (1) hardware designs for the computational node along with instructions for building a prototype device using off-the-shelf components, including performing all of the hardware modifications; (2) a PCB design with all information required to manufacture them commercially; (3) firmware code along with more detailed documentation about the organization and functioning of the firmware; and (4) data and scripts to reproduce calculations presented in the Case Study Application section of this paper.

## 2. System Description

The CIWS computational node was designed to collect, process, and transfer high temporal resolution water use data on existing residential analog water meters and to meet the following requirements: (1) operation on top of existing, analog meters without affecting the function of the meter (i.e., recording data for a water utility’s monthly billing purposes); (2) autonomous operation for at least two weeks without supplemental power, including data collection, processing, and transfer; (3) simplicity of deployment and use with an easily operable user interface; (4) generalized support for computations to be performed (e.g., execution of any data processing code); and (5) output data and computed results in accessible, platform-independent formats without requiring visits to deployment sites for manual data downloading. The hardware and software of the CIWS computational node are open source and can be customized to fit specific research needs, which means that the CIWS node is an open and customizable platform for collecting and processing high temporal resolution water use data at the edge.

The CIWS computational node adopts a double processor architecture to achieve both low power consumption and computational capabilities. The first processor is a low-power Arduino microcontroller that continuously collects high temporal resolution water use data and temporarily stores it within an electrically erasable programmable read-only memory (EEPROM) chip. The second processor is a Raspberry Pi single-board computer that reads the data from the EEPROM chip, writes the data to its own file system, and then executes any code that has been designed to process the raw data (e.g., identifying individual end uses, classifying them, and then transferring raw, event, and/or other summary data to a remote server). Arduino is an open-source hardware and software platform with an AVR single chip microcontroller [25]. Raspberry Pi is a small, single-board computer that uses an open-source distribution of the Linux operating system [21]. As it is always collecting data, the Arduino platform is always powered. Given its higher power consumption, the Raspberry Pi computer is only powered during periods when computations are to take place.

We developed two prototypes of the computational node. We iterated on the first prototype using off-the-shelf components, including an Arduino Pro microcontroller and a Raspberry Pi computer, to perfect our design. The second prototype consists of a PCB that integrates all of the components into a single “hat” that can be interfaced directly with a Raspberry Pi computer’s pin header. The PCB hat includes only those components needed by the computational node to function and was intended to make the computational node easy to manufacture. For example, we included the ATmega328P microcontroller [26] used by the Arduino Pro in the PCB design without the unnecessary peripherals. Given this, we use the term “microcontroller” in the sections that follow to refer to either the Arduino Pro in our off-the-shelf prototype or the ATmega328P in our PCB prototype.

### 2.1. Principle of Functioning

The CIWS computational node uses the same methods for measuring flow through magnetically-driven water meters as the CIWS datalogger. Here, we provide a brief description for completeness, but more specific details are provided by [7]. Both the CIWS datalogger and CIWS computational node were designed to measure and record water flow through magnetically-driven, residential water meters. Many meters of this type use a nutating disc, rotating impeller, or other similar element to measure water flow using the positive displacement principle. When water flows through a meter’s fixed volume measurement element, the nutating disc or rotating impeller is actuated. A nutation of the disc or rotation of the impeller corresponds to a fixed volume of water. The rate of nutation or revolution is proportional to the flow rate, and the count of nutations or revolutions is recorded using a magnetically-driven register. A magnet inside the meter’s register is paired with a spinning magnet attached to the measurement element inside the meter’s sealed housing. These paired magnets rotate together, and the revolutions of the magnet are counted by the meter’s register to determine flow rate and volume. The CIWS computational node uses a magnetometer sensor mounted to the outside of the meter’s housing to measure water flow by counting and recording the number of times the magnet inside the meter rotates.

The computational node differs from the CIWS datalogger in how it records the raw pulse data. Instead of immediately writing pulse count data to an SD card connected to the microcontroller, the microcontroller stores data within an EEPROM chip. As the microcontroller is always powered and collecting data, it is responsible for switching power to the Raspberry Pi computer, which triggers the Raspberry Pi computer to boot, read data from the EEPROM chip, and then run any computational or data transmission code.

The software of the CIWS computational node is comprised of five main modules: LoggerShell_CLI.py, logger.c, LoggerAutoRun.py, piHandler.py, and arduinoHandler.py (see Section 2.4 for more details on the software running on the computational node). LoggerShell_CLI.py is the command line interface for all of the datalogging functionality. logger.c is the module designed for communication with the attached AVR-based datalogger. LoggerAutoRun.py is the autonomous functionality of the node executed every time the Raspberry Pi computer is powered on. piHandler.py is the computational module of the node responsible for handling data processing, data transfer, and data storage functions used by the Raspberry Pi computer. arduinoHandler.py is a wrapper module for logger.c responsible for handling functions used to communicate with the Arduino.

User-created computational code, written in Python, can be imported into the piHandler.py module. All computational code is passed the name of the file that contains the raw data. The computational code reads the data, performs computations, and writes its output to a new file and returns the name of this new file. When all computations are complete, the raw data file and all computation files are, depending on the settings set by the user, stored on the Raspberry Pi computer and sent to a remote server. To send data to a server, the user needs to fill in the upload_url, upload_token_url, and client_passcode strings in the piHandler.py module. When these parameters are filled out, the device is capable of sending data using the Python requests HTTP module.

### 2.2. Hardware

**Figure 1:**
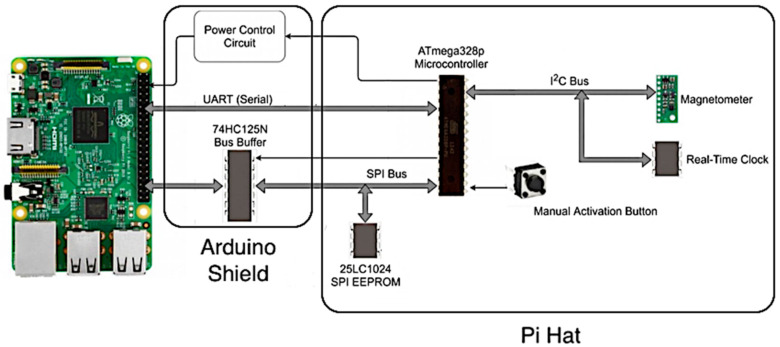
The CIWS computational node hardware architecture, including a Raspberry Pi computer, an Arduino datalogging shield, and a custom-designed Pi hat.

The CIWS computational node’s main components include a microcontroller, an EEPROM flash storage chip, a magnetometer sensor, a Raspberry Pi computer, a bus buffer, a power control circuit, a real-time clock, and a manual activation button (Figure 1). In brief, the microcontroller collects pulse data and stores it on the EEPROM chip. The Raspberry Pi computer, when powered by the microcontroller, reads the pulse data from the EEPROM, processes the data, and stores the data and any computational results in files on its Micro SD card. The microcontroller and Raspberry Pi computer communicate with each other using a universal asynchronous receiver-transmitter (UART) serial connection rather than over the serial peripheral interface (SPI) bus, which is only used for reading and writing data to the EEPROM in this design. The CIWS computational node’s main components are described in detail in the following sections.

We implemented our off-the-shelf prototype using an Arduino datalogging shield and a custom shield designed to interface with a Raspberry Pi computer. This dual system enables low power data logging by the microcontroller and periodic, resource-heavy computations on the Raspberry Pi computer. During a majority of the device’s on-time, the Raspberry Pi computer is powered off. The microcontroller is always powered on, but we made several modifications to reduce power consumption. Only timers and peripherals necessary for logging data using the microcontroller are maintained. Modifications included disabling and enabling peripherals such as UART, SPI, and two-wire interface (TWI), as needed. The Raspberry Pi computer is directly connected to the EEPROM chip, but has a buffer in line to ensure there is no interference by the Raspberry Pi computer while the microcontroller is writing data. The Raspberry Pi computer and microcontroller communicate with each other using UART and two general purpose input/output (GPIO) pins.

#### 2.2.1. Data Logging Components

The CIWS computational node’s data logging components include an Arduino microcontroller, an EEPROM flash storage chip, and a magnetometer sensor. The Arduino microcontroller implemented in the design is the ATmega328p 8-bit AVR core microcontroller [26]. The microcontroller is primarily responsible for collecting raw data from the magnetometer, detecting pulses in the data, storing pulse counts to the EEPROM, controlling power to the Raspberry Pi computer, controlling the Raspberry Pi computer’s access to the EEPROM, communicating with the Raspberry Pi computer, and generating timestamps for each recorded data value using information from the real-time clock (RTC). The EEPROM flash storage is a 25LC1024 SPI EEPROM chip [27] that can store 128 kB of data from the microcontroller. The data format stored in the chip is listed in Table 1.

The magnetometer used in our prototype is an LIS3MDL magnetometer by ST Microelectronics [28]. The LIS3MDL we use is mounted to a board and sold by Pololu [29]. The LIS3MDL magnetometer has several configurable sample rates. The sample rate used in our design is 560 Hz. When the LIS3MDL signals that the data are ready, the data are read by the microcontroller using the I2C serial bus and are processed and stored on the EEPROM chip. For full details on the data logging components, see [7].

#### 2.2.2. Raspberry Pi

The Raspberry Pi computer used in the system is the third-generation Model B version [30]. The Model 3B is based on a 1.2 GHz Broadcom BCM2837, ARM Cortex-A53 processor. We used the default Raspbian operating system to run the code we developed on the Raspberry Pi computer. We chose a Raspberry Pi computer for this application because it provides a fully functional operating system that has all the features of a computer, including a processer, random access memory (RAM), ability to run sophisticated computer code, communications capabilities, and a file system for managing files. The Raspberry Pi computer is responsible for retrieving data recorded by the microcontroller and then performing any data processing, which includes writing the data to the Raspberry Pi computer’s file system and any computations required by the user. The Raspberry Pi computer is also responsible for transmitting data over the Internet to a remote server. In addition to this technical functionality, the Raspberry Pi computer implements a user interface for the datalogger (see Section 2.4). Via the user interface, the user settings are communicated to the microcontroller, and information from the microcontroller is communicated to the Raspberry Pi computer and then to the user.

#### 2.2.3. Bus Buffer

Bus buffers are used to provide a sufficient drive capability to pass signals and enable communication between several devices over the same bus. Since the computational node has two master devices (the Raspberry Pi computer and the microcontroller) trying to communicate with the same servant device (the EEPROM), we used the bus buffer to make sure data are not corrupted when both the Raspberry Pi computer and the microcontroller try to communicate with the EEPROM. The bus buffer was implemented using a 74HC125N buffer chip [31]. We used the bus buffer to connect the SPI bus on the Raspberry Pi computer, the microcontroller’s SPI bus, and the EEPROM chip together. The buffer is controlled by the microcontroller and controls data transmission from the EEPROM to the Raspberry Pi computer. When the buffer is activated by the microcontroller, the Raspberry Pi computer is connected to the EEPROM chip. When the buffer is deactivated by the microcontroller, the Raspberry Pi computer is disconnected from the EEPROM chip. This permits the SPI bus to be used while the Raspberry Pi computer is off and is necessary because driving the I/O pins of the Raspberry Pi computer while it is powered off can cause damage to the computer.

#### 2.2.4. Power Control Circuit

**Figure 2:**
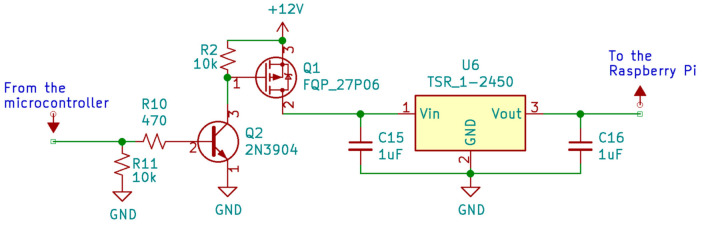
Power control circuit of the CIWS computational node.

The power control circuit switches power on and off to the Raspberry Pi computer and is controlled by the microcontroller. Its design required mediating across the different power levels of the power supply (5 V or greater), the Raspberry Pi computer (5 V), and the microcontroller (3.3 V). In the current design, the Raspberry Pi computer is powered on once per day at midnight. When the Raspberry Pi computer is powered on, data are copied from the EEPROM to the Raspberry Pi computer’s memory, which are then processed, and the results are transferred to a remote server. When data transfer is finished, the Raspberry Pi computer turns itself off, and the microcontroller cuts power to the Raspberry Pi computer. The power control circuit we used to enable power switching between the Arduino microcontroller and Raspberry Pi computer is shown in Figure 2. This diagram is part of a larger design schematic for the computational node that is available in the project’s GitHub repository (see the Data Availability section).

In Figure 2, R10 and R11 are the Arduino Pro’s resistors, Q1 and Q2 are transistors, C15 and C16 are capacitors, U6 is a power regulator, Vin is the input voltage, Vout is the output voltage, and GND is the ground reference point. The power control circuit can be powered by any battery with a voltage equal or larger than 5 V. The voltage regulator U6 is the component responsible for converting the battery voltage down to 5 V for the Raspberry Pi computer. The two capacitors C15 and C16 smooth out the power supply voltage so that the Raspberry Pi computer does not experience sharp changes in voltage away from normal operating levels at 5 V. The transistor Q1 sits between the battery (>5 V) and U6 and acts as a switch. When it is turned on, U6 will output 5 V. When Q1 is off, U6 will have no input voltage, and will, therefore, give no output voltage. To turn Q1 on, the ‘gate’ pin (labeled ‘1’) must be set to ground. To turn Q1 off, the ‘gate’ pin must be set to the supply voltage. The microcontroller can set the pin to ground with no issues; however, the microcontroller by itself can only set a pin to 3.3 V because its supply voltage is 3.3 V. To remedy this problem, a second transistor (Q2) is used. Q2 is a different kind of transistor and works differently than Q1. To turn on Q2, the electric current must flow into it through pin 2, the ‘base’ pin. To turn off Q2, no current can flow. This process is controlled by the microcontroller. When Q2 is ‘off’, the gate pin on Q1 is connected to the supply voltage, which ensures that Q1 is ‘off’ and cannot conduct. This then turns off the voltage regulator U6, which cuts the power supply to the Raspberry Pi computer. When Q2 is ‘on’, the gate pin of Q1 is no longer connected to the supply voltage. Instead, it is connected to ground. This allows Q1 to conduct and turn on the voltage regulator U6, which supplies power to the Raspberry Pi computer.

#### 2.2.5. Real-Time Clock

In order to accurately control the sampling interval and to record timestamps associated with each raw data value, the system uses an RTC. We chose to use a PCF8523 RTC manufactured by NXP Semiconductor [32]. This RTC is already present on the Arduino datalogging shield and was, thus, a simple prototyping choice for the RTC. The PCF8523 also communicates with the microcontroller using I2C and shares the I2C bus with the LIS3MDL magnetometer. The RTC is used by the microcontroller for sample interval timing and timestamp generation. The RTC signals the microcontroller whenever a specified data recording interval has passed, and the microcontroller then counts up the pulses detected by the magnetometer sensor and stores that value in the EEPROM. The date/time is also read from the RTC when this interval has passed, from which the microcontroller creates a timestamp.

#### 2.2.6. Manual Activation Button

The manual activation button is used to start up the Raspberry Pi computer manually. This component is required to enable a user to interact with the Raspberry Pi computer on demand rather than waiting for it to be turned on automatically by the microcontroller. When the manual activation button is pressed, the Raspberry Pi computer powers on and waits for the user to log in via a terminal. The user can then log in to the system to view files, start/stop a logging session, view water flow data, and perform other tasks available through the user interface (see Section 2.4).

Table 2 lists all components, sources, and approximate costs per unit at the time of this writing to build a CIWS computational node using off-the-shelf components. The cost to build a node is approximately $199.47 with pricing that varies depending on the number of components purchased. Some parts, including cables and connectors, are only available in quantities larger than what is needed for a single node. The costs presented in Table 2 were estimated after purchasing the materials needed to build three nodes. Specific part numbers and a URL link to each vendor are available in the project’s GitHub repository.

#### 2.2.7. Printed Circuit Board Design

**Figure 3:**
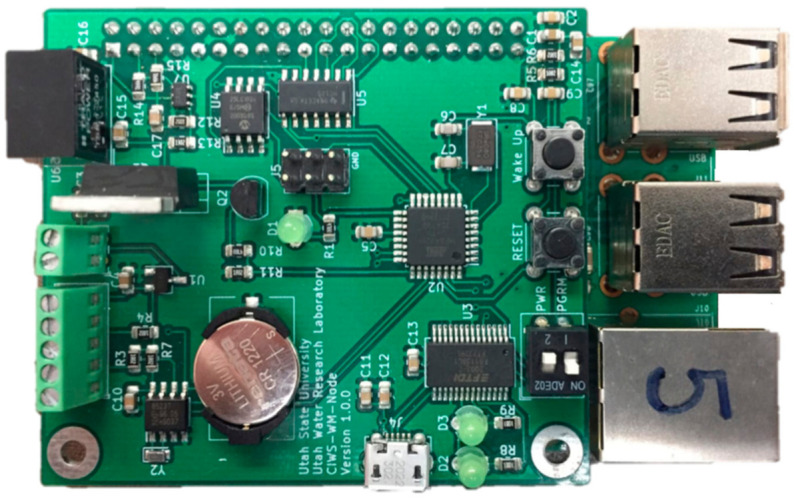
Printed circuit board implementation of the CIWS computational node. This board is designed to mount directly to the pin header of the Raspberry Pi computer (shown underneath the PCB) and includes all of the hardware components on a single board.

In an attempt to reduce the time and effort required to manufacture the CIWS computational node using off-the-shelf components, we translated the design for our prototype computational node into a PCB design. The PCB design includes all of the hardware components required to produce a hardware “hat” that interfaces directly with the pin header on a Raspberry Pi 3B computer. We sent the PCB design files to the PCBWay PCB manufacturing company [33] for production and assembly and ordered and tested a small run of five devices to verify their functioning. We successfully tested the PCB devices in a laboratory setting using the testing procedure described in Section 3. The total cost for manufacturing and assembling a PCB device (Figure 3) was $61.90 USD, which included manufacturing of the PCB and placing of all of the components to create a finished product. The manufacturing cost can be reduced with bulk orders for a larger number of devices. The information required to manufacture the Computational node PCB, including schematics showing connections between all of the parts, Gerber design files with configuration parameters, aperture definitions, coordinate information for the location of parts, and a list of the materials required, is publicly available in the project’s GitHub repository.

### 2.3. Microcontroller Firmware

Similar to the CIWS datalogger, the firmware for the CIWS computational node is organized using the C-like Arduino programming approach and was developed within the Arduino Interactive Development Environment (IDE) [34]. For each of the libraries that were developed for the CIWS computational node (System State, Store New Record, Real-Time Clock, and System State), the source code and detailed documentation of the functions developed within each library are available in the project’s GitHub repository. In the sections that follow, we describe the libraries developed specifically for the CIWS computational node and their main functions. Along with the newly-developed libraries, additional libraries developed originally for the CIWS datalogger [7] were used, including the detectPeaks, magnetometer, and powerSleep libraries. For completeness, these libraries are included in the GitHub repository for the CIWS computational node, but the reader is directed to [7] for details regarding the functionality each contains.

**Figure 4:**
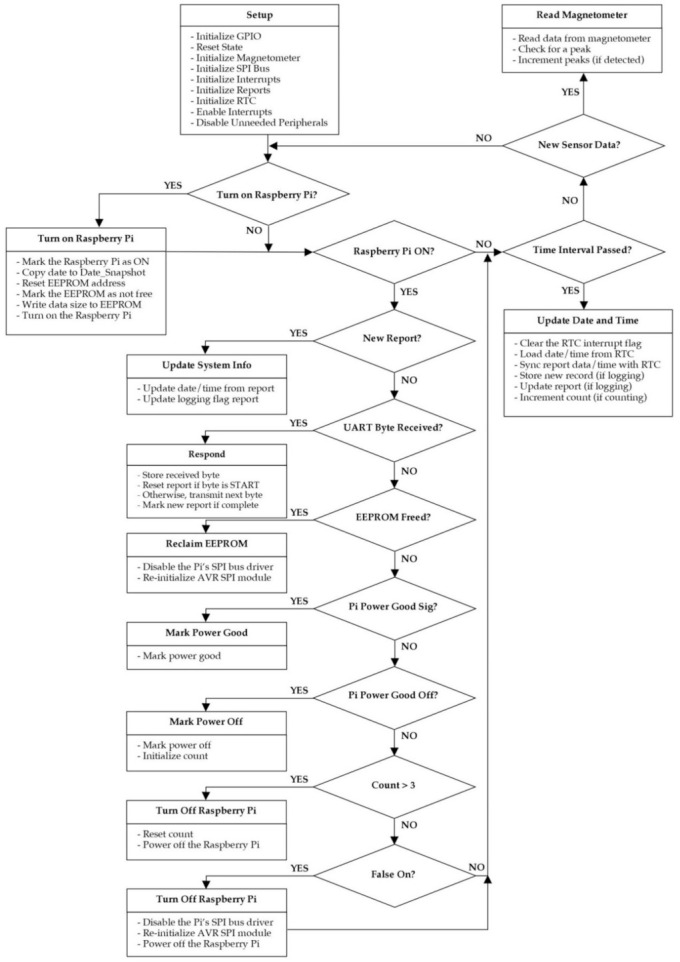
CIWS firmware architecture.

The main firmware file that operates and controls the CIWS computational node, “Computational_Firmware.ino”, calls all of the libraries mentioned above. It contains four functions: (1) setup(), (2) loop(), (3) INT0_ISR(), and (4) INT1_ISR(). A flow chart describing the firmware is shown in Figure 4. As with most microcontroller programs, the setup() function is called once when the device is powered, and the loop() function is called repeatedly until the microcontroller is reset. The functions INT0_ISR() and INT1_ISR() are interrupt service routines that are executed when an event in hardware occurs. These functions manage the retrieval of new data from the magnetometer sensor and the RTC. As the CIWS computational node uses the same sensor measurement and observation timing as the original CIWS datalogger, both functions were adopted as-is from the CIWS datalogger firmware [7]. Table 3 lists the main objective of the four functions that comprise the firmware of the CIWS computational node and are included in the Computational_Firmware.ino file.

#### 2.3.1. System State Library

The system state library defines two C/C++ structures: State and SignalState. State keeps track of several important values (Table 4). When the State structure is initialized, the pulse count is set to zero, the record number is set to one, and the Boolean flags are initialized to false. The SignalState structure keeps track of values used for processing the magnetometer signal, including the input signal from the magnetometer, direct current (DC) removal filter pole, output signal from DC removal filter pole, and software-based Schmitt trigger.

#### 2.3.2. Real-Time Clock Library

The RTC library defines a list of hexadecimal addresses and a date/time structure for the RTC’s registers in the microcontroller, which holds the current year, month, day, hour, minute, and second. Table 5 lists the functions defined in the RTC library and their main objective.

#### 2.3.3. Store New Record Library

The storeNewRecord library defines three functions responsible for storing data in the EEPROM chip, including writeDataSize(), writeDateAndTime(), and storeNewRecord(). The function writeDataSize() is used to write the current number of records to the EEPROM chip. This function is called when the user presses the activation button, or once a day at midnight, so that the number of records on the EEPROM chip is written where the Raspberry Pi computer can find it and so that the Raspberry Pi computer knows exactly how many bytes to read from the EEPROM chip. The function writeDateAndTime() is used to write a timestamp to the EEPROM chip. This function is called when the first data record is written to the EEPROM chip. The timestamp is written to the address range 0x003–0x008 of the EEPROM chip. This function is called by storeNewRecord(). The function storeNewRecord() is used to store a data record to the EEPROM chip.

The default storage for new data records is the EEPROM chip. However, in some instances, data may be temporarily stored in an array allocated in the microcontroller’s volatile, static random-access memory (SRAM). As long as the EEPROM chip is not being used by the Raspberry Pi computer, all data records bypass the array and are stored directly in the EEPROM. If the EEPROM chip is being used by the Raspberry Pi computer, then the data record is stored in the array. As soon as the Raspberry Pi computer releases the EEPROM back to the microcontroller, the data in the array are appended to the end of existing data records section of the EEPROM chip. Once this process is complete, the array index resets to zero.

**Figure 5:**
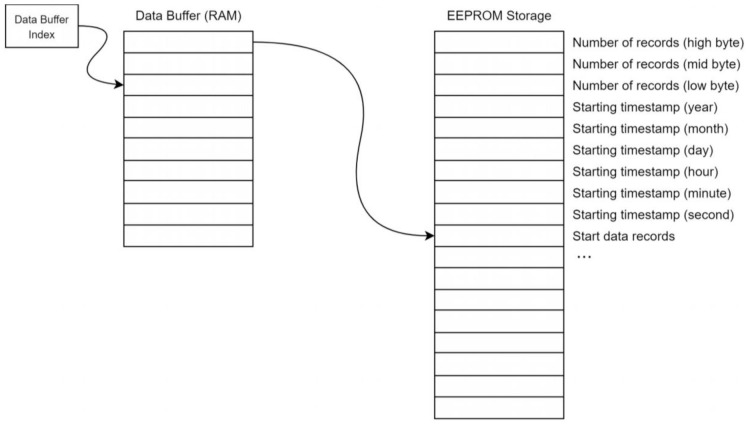
CIWS computational node record storage architecture.

Unlike the CIWS datalogger project, an individual data record consists of only one byte, which is the number of pulses detected within the time interval of the record. The Raspberry Pi computer fills in the record number and timestamp when it reads the data from the EEPROM. In addition to storing the current record to the EEPROM chip, the storeNewRecord() function also checks for records in the array waiting to be written to the EEPROM. If there are records and the EEPROM is free, they are written to the EEPROM first, followed by the current record. If the EEPROM is being used by the Raspberry Pi computer, then the current record is instead written to the secondary data buffer. If the Raspberry Pi computer has not freed up the EEPROM when this function is called and the secondary data array is full, the program sets a flag in the system state structure, RPiFalseON, which indicates to the program that the Raspberry Pi computer has, for some reason, failed to release the EEPROM chip. The Raspberry Pi computer will then be shut down by cutting the power, and data in the secondary buffer will be appended to what is currently in the EEPROM. Unfortunately, this solution creates the potential for data loss if the Raspberry Pi computer freezes before releasing the EEPROM, but is required as a last resort to ensure that the computational node can continue operating in the event that the Raspberry Pi computer encounters a fatal problem. Figure 5 illustrates the record storage architecture and the data format for the EEPROM.

The number of records is stored in the first three bytes of the EEPROM (addresses 0, 1, and 2). The byte at address zero is the most significant byte or high byte. The byte at address one is the middle byte, and the byte at address two is the least significant byte. This number corresponds to the number of data records after the timestamp data in the EEPROM. The next six bytes of the EEPROM hold the starting timestamp. This timestamp is the timestamp associated with the first data record in the EEPROM and is the only timestamp stored in the EEPROM. Because the data records are stored based on timing from the RTC, the timestamps for each record can be determined from the original timestamp by the Raspberry Pi computer. The timestamp is composed of six bytes, with one byte per field. The fields are year, month, day, hour, minute, and second. The EEPROM is read and written to using an SPI bus, which is a common peripheral on many microcontrollers, including the ATmega328p we used. A brief description of the transaction with the EEPROM chip is provided here. Further information can be found in the 25LC1024 datasheet (Microchip document DS22064D) [35].

Every time the microcontroller writes to the EEPROM, it must enact two SPI transactions: (1) send a write-enable instruction and (2) send the data to be written. Sending the write-enable instruction consists of sending the single byte 0x06. Writing this byte enables the EEPROM to accept a write instruction. This byte must be sent as a separate SPI transaction before any data writing can be attempted. There is no response from the EEPROM when the write-enable instruction byte is sent. After the write-enable instruction byte has been sent to the EEPROM, the EEPROM is ready to accept the data to be written. The maximum number of data bytes that can be written in a single transaction is 256, as long as all 256 bytes reside on the same page (memory block) in the EEPROM chip.

#### 2.3.4. Communication Library

The Communication library defines several functions used for communicating between the EEPROM chip and the Raspberry Pi computer. The functions provide an interface to SPI bus transactions, UART transactions, powering the Raspberry Pi computer on and off, and updating system information based on data in a “report”. Table 6 lists the functions defined in the Communication library and their main objective.

### 2.4. Software

The microcontroller powers the Raspberry Pi computer when the user presses a physical activation button, or once a day at midnight. The Raspberry Pi computer must then autonomously read, process, and store the EEPROM data with no intervention from the user. The Raspberry Pi computer must also power itself back off again. All of this is done by the autonomous functionality script of the node (LoggerAutoRun.py). The script was implemented in Python and is run at startup from an rc.local command. rc.local is a file in the Linux directory whose commands are run at startup. In the node’s design, the LoggerAutoRun.py is executed every time the Raspberry Pi computer is powered on and boots. If the power-on event occurs at the scheduled time at midnight, the script assumes that the power-on was automatic and the Raspberry Pi computer is shut down after it reads data from the EEPROM and performs any computations needed. Otherwise, the script assumes that the user powered on the Raspberry Pi computer via the activation button, and the Raspberry Pi computer is not shut down.

The LoggerAutoRun.py script calls a set of functions in a module called arduinoHandler.py, which was developed to enable interactions between the Raspberry Pi computer and the microcontroller. For example, upon being powered, the Raspberry Pi computer automatically runs the LoggerAutoRun.py script, which calls the setPowerGood() function from the arduinoHandler.py module. The setPowerGood() function sets the Raspberry Pi computer’s general purpose input output (GPIO) 25 pin high, which alerts the microcontroller that the Raspberry Pi computer has successfully powered on. The LoggerAutoRun.py script then calls the writeEEPROMToFile() function from the same arduinoHandler.py module to create a file to hold the data stored in the EEPROM by the microcontroller, calls a SPI transaction to read the data from the EEPROM chip, and copies the data to the data array in the Raspberry Pi computer’s memory.

Copying data from the EEPROM is initiated by calling the reportSwap() function from the arduinoHandler.py module, which sends a START byte to the microcontroller. Sending a new START byte will always initiate a new data copy, even if one is in progress. Data transaction from the EEPROM to the Raspberry Pi computer’s memory is done once per day to conserve power and create data files containing one day of uninterrupted data, which is convenient for some data processing steps (e.g., daily summaries). Data are temporarily saved on the EEPROM until it is full. When the EEPROM memory is full, newly recorded data points are written and saved over the oldest data points.

While reading from the EEPROM, the Raspberry Pi computer sets its GPIO 24 pin high using the setRomBusy() function, which alerts the microcontroller that the Raspberry Pi computer is reading the EEPROM. After copying all data from the EEPROM to the Raspberry Pi computer, the Raspberry Pi computer calls the writeEEPROMToFile() function to translate it into a comma separated values (CSV) file and optionally save it within the file system on the Raspberry Pi computer’s Micro SD card memory. Both setRomBusy() and writeEEPROMToFile() are called from the arduinoHandler.py module.

Once the data copy from the EEPROM is complete, the Raspberry Pi computer is free to run any computational code that the user requires. We describe a case study application for end use disaggregation and classification in the section that follows, but we designed the firmware for the node to enable execution of any computational code. Computational code files are handled using the dataAnalysis() function, which loops through all computational code files in the piHandler.py module, executes them, and returns the outputs of each computational code file as CSV files.

For each raw data file and processed data file returned by the writeEEPROMToFile() and dataAnalysis() functions, respectively, the Raspberry Pi computer either saves them in the software data directory in the Raspberry Pi computer’s file system, or transfers them to a remote server based on the data transfer configuration set by the user. If the device configuration is set to save the data on the Raspberry Pi computer, they are saved in the savedData directory of the Raspberry Pi computer. If the device configuration is set to transfer the data to a remote server, the send() function is initiated. The send() function is implemented within a Python module called PiHandler.py.

All data transfers from the node to a remote server are handled by sending files using HTTP POST requests to a data posting service (DPS) hosted on the remote server (for more details of the server software, see [36]). The standard Python requests library was used to implement the creation of HTTP POST requests to transfer the data from the node to the web server. To enable sending data to a server, the user needs to set the upload_url, upload_token_url, and client_passcode strings in the piHandler.py module. The upload_url field contains the database server hostname or IP address. The client_passcode is the password for a user with permission to write data to the remote server. The upload_token_url is an authentication key used to authenticate upload requests. When the Raspberry Pi computer sends an HTTP POST request containing data files (raw data and/or processed data) to the server, the requests are received and handled by the DPS. The DPS authenticates HTTP POST requests using the token supplied by the user (upload_token_url, for more details see [36]). 

A flag set by the user in the initial configuration of the device is used to determine which files will be transferred to a remote server. The user can modify the data transfer flag to decide which files to transfer to the remote server through the user interface shell using the set-transmission command. When a flag value of “1” is set, only unprocessed raw data files are sent to the server. When a flag value of “2” is set, only files resulting from computations are sent to the server. When a flag value of “3” is set, both raw data files and files resulting from computations are sent to the server. Data transfer uses an all-or-nothing protocol; meaning when data transfer is initiated, all data consistent with the flag setting are transferred. Data transfer can be accomplished using the Raspberry Pi computer’s integrated WiFi or via other attached radios (e.g., cellular).

Once all data computations and data transfer are finished, the Raspberry Pi computer sets both GPIO 24 and GPIO 25 pins to low by calling the setPowerOff() and setRomFree() functions from the logger.c module. Setting the GPIO 24 pin low sends a signal to the microcontroller that the Raspberry Pi computer has finished reading the EEPROM. Setting the GPIO 25 pin low sends a signal to the microcontroller to power the Raspberry Pi computer off. The microcontroller will cut power to the Raspberry Pi computer roughly 12 s after the signal is received.

**Figure 6:**
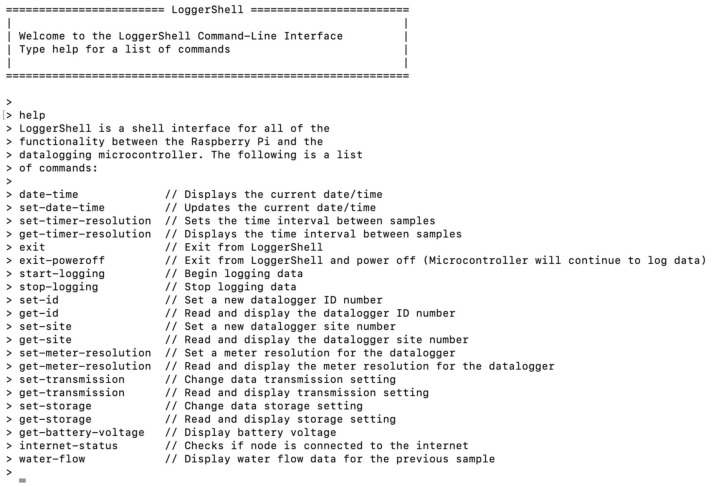
CIWS computational node user interface help menu showing available functions.

We developed an interactive, command line shell interface (LoggerShell_CLI.py) in Python as a user interface to all of the functionalities of the computational node, including the Raspberry Pi computer and microcontroller. The user interface allows users to execute basic functions needed to configure and operate the computational node, along with managing and retrieving processed and unprocessed data files. This includes configuring the device to work with different water meter brands and sizes. The user interface operates on the Raspberry Pi computer and can be accessed through any serial terminal emulator wirelessly via WiFi, or through a direct Ethernet connection. To access the user interface via the interactive shell on the serial terminal, the power button on the datalogging shield is clicked first, which will power the Raspberry Pi computer and cause it to boot. Once powered on, a serial connection can be made to the Raspberry Pi computer. By default, the LoggerShell_CLI.py Python script automatically displays the help menu, and the set of commands listed in Figure 6 will be accessible.

## 3. Calibration

Laboratory experiments were conducted to ensure that the computational node can accurately measure, process, and transfer water use data at different water flow rate conditions and for different meter types and sizes. Two different water use scenarios were tested in laboratory experiments to examine the data collection accuracy of the device. In both experiments, we pumped water through test meters at multiple flow rates ranging from 0 to 75 LPM for 30 min. In the first experiment, we used a flow controlling valve to pump uninterrupted water flow through the meters while increasing the flowrate every 10 min. This enabled us to test the accuracy of data collection over the range of flowrates expected for residential water meters. The second experiment was similar to the first, but we used the flow controlling valve to interrupt the flow between each flowrate increase. This enabled us to ensure that the computational node accurately measures flow across multiple, discrete events as expected within residential homes. We manually read the manufacturer’s register for each meter before and after each run. We calculated the volume of water used in each run as the difference in manual register readings. We calculated the volume of water logged by the computational node on each meter as the number of pulses recorded by the node multiplied by the pulse resolution of each meter. We then compared the volume registered by the meter’s register with the volume registered by the computational node to ensure that the computational node accurately recorded water flow.

In both experiments, a CIWS computational node was installed on top of a 1-inch Bottom Loading (BL) Master Meter and another on a 5/8-inch Master Meter of the same model. Meter sizes are reported in inches consistent with how these meters are sold in the U.S. The water use volume registered by the meter’s register and the CIWS computational node on the 1-inch meter were 756.8 L and 756.39 L, respectively, for the first experiment and 618.08 L and 625.12 L, respectively, for the second experiment. A maximum percent error of 1.35% was observed across both experiments. For the 5/8-inch meter, the water use volume registered by the meter and the CIWS computational node were 757.35 L and 762.31 L, respectively, for the first experiment and 621.60 L and 633.24 L, respectively, for the second experiment. A maximum percent error of 1.87% was observed across both experiments. Table 7 and Table 8 show the volumes read by each meter’s register, the volumes captured by the corresponding CIWS computational node, and the percent error for each step for the meters tested in the laboratory.

## 4. Case Study Application

As a case study for demonstrating the node’s data collection and computational functionality, we developed an application for automatically identifying and classifying end use events from raw water trace data recorded by the node on a residential water meter. The node was configured to record raw water use data with a four second recording interval. We then used the CIWS disaggregator algorithm designed by [24] to process the raw water use data to produce classified events. We used the Raspberry Pi computer’s terminal and the package installer for Python (pip) to install all of the dependencies and Python libraries required by the CIWS disaggregator algorithm on the Raspberry Pi computer. We then placed the CIWS disaggregator algorithm in the software working directory of the Raspberry Pi computer. Prior to deployment in the field, we tested and verified the computational and data transfer capabilities of the node using test data files from a prior study [24]. We manually placed the test dataset in the data working directory of the Raspberry Pi computer, manually executed the CIWS disaggregator algorithm by calling the dataAnalysis() function, and then manually executed the data transfer by calling the send() function.

Once we verified that the node was working correctly in a laboratory setting, we then ran a field deployment of the node. In the field, we installed the node on the water meter for a home in the city of Providence, Utah, USA between 17 January 2021 and 11 February 2021 to evaluate its performance under field conditions. We installed the node with a new, fully charged, 12 V, 10 Ahr battery and ran data collection, event disaggregation, and data transfer to a remote server until the battery failed. This deployment enabled us to test the power consumption of the device and estimate the length of autonomous operation we could expect without an external power supply. During the field deployment, we configured the Raspberry Pi computer to connect to the homeowner’s WiFi network using its integrated WiFi capability. We also set the device to execute the CIWS disaggregator algorithm, store both raw data and classified event files on the Micro SD card on the Raspberry Pi computer, and transfer both sets of files to a secured server at midnight every day. In the case of a WiFi network failure, we designed the device to store the files within the Raspberry Pi computer’s file system on the local Micro SD card. When the WiFi connection is restored, all files stored on the Raspberry Pi computer’s Micro SD card are transferred to the server.

The remote server to which files were transferred consisted of an instance of the CIWS cyberinfrastructure described by [36], which includes a data posting service that was specifically designed to authenticate and accept data files posted to the server via HTTP POST requests from devices like the computational node. The remote server was implemented on an Ubuntu Linux virtual machine hosted within Utah State University’s Enterprise Data Center. While it is beyond the scope of this paper to describe the details of the larger cyberinfrastructure needed to manage the data created by a network of operational nodes, readers are referred to [36], where we performed scalability testing to investigate the performance of the CIWS cyberinfrastructure and showed that even a modestly provisioned server could robustly handle HTTP POST requests from hundreds of active nodes submitting data at the same time.

### 4.1. Data Output

For our case study deployments, the CIWS computational node output two comma-separated values (CSV) files per day: one for the unprocessed, high-resolution water use data and another for the classified water end use events output by the CIWS disaggregator algorithm. The first three rows in the raw data file are reserved for a standard metadata header that includes a site number at which the node is deployed, a unique identifier for the node, and the meter pulse resolution where the device is installed, i.e., the volume of water corresponding to each magnetic “pulse” recorded by the meter, see [7]. These values were set using the node’s user interface. The fourth row serves as a header for the data and has three fields: Time, Record, and Pulses. The Time field contains the date and time values at which individual observations of water use were recorded. The Record field is a sequential numerical ID used to keep track of the number of observations logged. The Pulses field is an integer number that corresponds to the number of pulses registered in a time interval.

**Figure 7:**
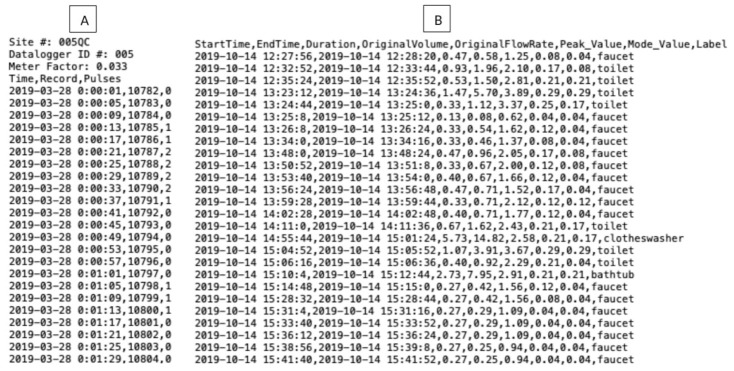
Sample output from the CIWS computational node. Panel (**A**) shows the raw data. Panel (**B**) shows the extracted and classified events from the raw data.

The classified water end use events file output by the CIWS disaggregator algorithm has eight fields: StartTime, EndTime, Duration, OriginalVolume, OriginalFlowRate, Peak_Value, Mode_Value, and Label. The StartTime field represents the start of an event and contains the date and time at which the water use volume transitions from zero to any positive value. The EndTime field represents the end of an event and contains the date and time at which the water use volume transitions back to zero. The Duration field is the elapsed time of the event in seconds. OriginalVolume is the summation of water use volumes of an event between its start and end times in gallons. OriginalFlowRate is the volume of an event divided by its duration and is recorded in gallons/minute. Peak_Value is the maximum rate of water flow within the event for any time step (gallons/minute). Mode_Value is the rate of water flow that appears most often within an event (gallons/minute). The Label field contains the water end use type of each event in the dataset output by the CIWS disaggregator algorithm. Figure 7 shows an example of two CSV files obtained from the CIWS computational node. Only a subset of records is presented.

### 4.2. Battery Life

During the first field deployment, we measured the voltage of the node’s battery before deployment and then monitored the battery’s discharge over the course of the field deployment until it was fully drained and the node failed. The discharge time was 26 days, indicating that the node could reasonably be used to collect, process, and transfer four-second temporal resolution and classified events data for over three weeks with no external power before the 10 Ahr battery has to be replaced.

**Figure 8:**
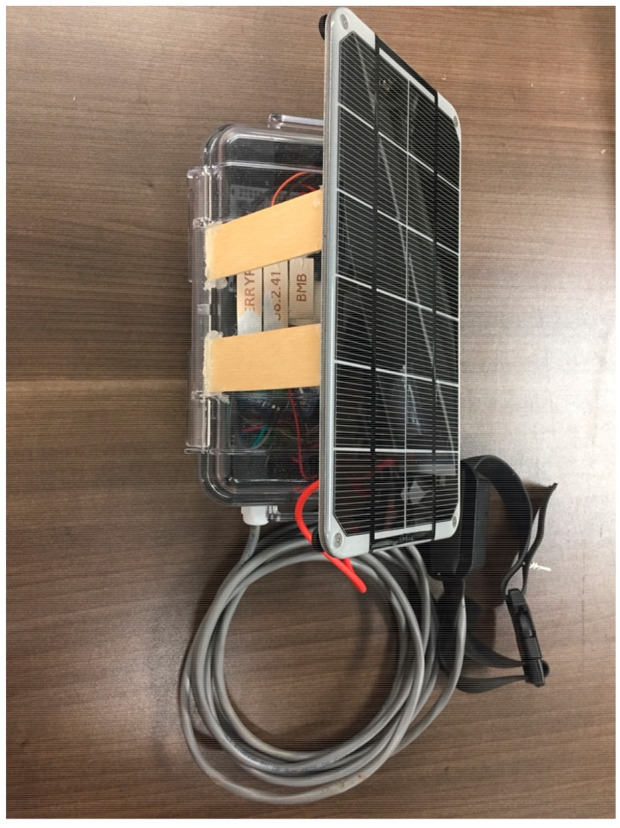
Solar-powered CIWS computational node prototype.

Where longer field deployments are needed, a higher capacity battery or a charge regulator connected to a solar panel can be used to enhance the lifespan of the device (Figure 8). In addition to the field deployment, we conducted quantitative power testing in the laboratory using a 4.4 Wh battery and a power regulator. We operated the node and monitored the battery voltage on a daily basis using a multimeter. The device’s power consumption was calculated by estimating the device’s full cycle power draw. The Raspberry Pi computer turns on once a day, so its power cycle consists of 24 h. When the Raspberry Pi computer is off, the device uses 0.04 W of power with a 12 V power supply. While the Raspberry Pi computer is running, 1.4 W are consumed at 12 V. The average power used by the device during a single cycle is dependent on how long the Raspberry Pi computer takes to perform its computations. For a device whose computations take 5 min to complete, the average power used by the device is 0.045 W. For a device whose computations take 10 min, it consumes 0.05 W on average.

In further testing to simulate potential field conditions with a small form-factor battery, we used Adafruit’s BQ24074 regulator with a 3.7 V, 4.4 Wh battery and observed 10 days of continual operation before the battery failed. The computational node regulates the input voltage to 5 V and 3.3 V for the Raspberry Pi computer and microcontroller, respectively. To boost the battery’s voltage from 3.7 V to a voltage that could be regulated down to 5 V we used an external Pololu U3V12F9 step-up voltage regulator to boost the voltage up to 9 V. Given the power requirements and considering the losses of the BQ24074 and U3V12F9, 10 days is a reasonable life expectancy for a 3.7 V, 4.4 Wh battery. A solar panel can be used to prolong the life of the device. The BQ24074 already supports connecting a solar panel. However, the practicality of adding a solar panel is highly dependent on the quality and quantity of sunlight available in the region. Put simply, a larger battery lengthens the lifetime of the device while a larger solar panel allows the device to recuperate quicker.

### 4.3. Accuracy

Evaluation of computational node performance in the field was based on multiple accuracy metrics, including volume accuracy, accuracy of event identification and classification, and accuracy of data transfer. To assess volume accuracy, we manually recorded the water use volume on the water meter’s register twice during the field deployment: once during the initial installation of the computational node and a second time two weeks after the installation. We calculated the total water use volume during the two weeks as the difference between the two manual readings. We then aggregated the pulses registered by the computational node for the same period of time and multiplied the total number of pulses by the pulse resolution of the meter to estimate the water use volume registered by the node. The volumes estimated from manual meter readings and from the computational node were 9402 L and 9459 L, respectively, with an error of 0.6%. This error is similar to the levels of error we observed within our laboratory tests. It is also less than the error threshold value of 5% used in our previous study [7], where we presumed that any error value within 5% was acceptable for the purpose of our study.

Evaluating the field accuracy of event detection and classification required collection of an additional dataset. During the field deployment period, we asked the home’s residents to manually label some water use events. For a subset of events in the home, the residents recorded the water end use type and the event start time. A total of 333 different water end use events were manually labeled during the field deployment as follows: 127 faucet events, 124 toilet flushes, 38 showers, 19 clothes washer events, 14 dishwasher events, and 11 bathtub events. The accuracy of the classified events was quantified as the fraction of water use events whose end use category was correctly predicted by the node when compared to the labels manually assigned by the home’s residents. The resulting classification accuracy of water end use events ranged from 100% for toilet, faucet, and clothes washer events to 64% for bathtub-filling events, which is consistent with the performance of the CIWS disaggregator algorithm reported in our earlier work, where classification was performed on a centralized computer after manually downloading the raw data from devices in the field [24]. The overall accuracy of the classification performed by the node was 98.4%. Given these results, we are confident that the CIWS disaggregator algorithm operated correctly on the computational node and classified events with the same level of accuracy achieved through manual downloading and post-processing.

During the period of our field deployment, we experienced no issues with data transfer from the computational node to a remote server located on Utah State University’s campus. The accuracy of data transfer was 100% and was quantified as the percentage of data transfer attempts that were successful. Successful attempts were verified by logging into the server each day and verifying that both raw data and classified event data files were uploaded successfully. We also verified that the transferred files were sent correctly by comparing the files on the server to the original files stored on the Raspberry Pi computer’s Micro SD card to ensure that they were the same.

### 4.4. Water Use

Using the data collection and computational capabilities of the CIWS computational node, we were able to identify and classify 1480 water use events retrieved from one residential household over the 26 days of our field deployment test, averaging 57 events per day and 14.2 events per capita-day. The average daily indoor water use of the studied household was 625.8 Lpd, and the average per capita indoor water use was 156.5 Lpcd. Compared to the per capita indoor water use for the state of Utah of 227.1 Lpcd estimated by the Utah Division of Water Resources [37], the studied household fell well below the estimate.

For the studied household, showers accounted for the largest volume of water use during the field deployment, followed by toilet flushing, clothes washer events, faucets, and bathtubs. Showers accounted for an average of approximately 34.6% of total indoor water use, toilets accounted for an average of 33.4%, clothes washer events contributed an average of 17%, faucet and dishwasher events contributed an average of 10.6%, and bathtub-filling events contributed an average of 4.3%.

With regard to utilization rate, we used the classified events to calculate the frequency of use for each end use type in the study home. Faucets were the most frequently utilized end use fixture at approximately 31.7 uses per day. Toilet flushing was the second most frequently utilized fixture at approximately 20.2 uses per day. Bathtub filling was the least frequently utilized indoor water end use, accounting for only 0.3 uses per day.

Using the capabilities of the computational node, we were able to produce directly on the node the information needed to perform a detailed water end use analysis. While we chose to transmit both the raw and disaggregated event data for testing purposes during our case study, the disaggregated event data calculated by the computational node is identical to what would be produced if the data were collected on the device, manually downloaded, and post-processed using a centralized computer. Thus, if the final use of the data is to examine classified end use events, the raw data need not be transferred to the server.

### 4.5. Limitations and Errors

An obvious limitation of the CIWS computational node observed during field deployment is related to communication. In Providence, UT, water meters are installed underground to prevent freezing during winter, and the depth of meter pits can exceed 0.5 m. The meter pit depth where the device was installed was approximately 1.5 m. WiFi signals are prone to attenuation by the meter pit casing and the soil surrounding the meter pit. To overcome this issue, and to ensure a reliable WiFi signal strength received by the device, we placed the magnetometer sensor on the meter underground, but placed the computational node in a weatherproof enclosure above ground. 

While we used the homeowner’s WiFi network for our field deployment testing, open WiFi networks are not ubiquitous, which may limit their use in larger deployments. Given this, we tested the data transmission capabilities of a Raspberry Pi computer connected to a Hologram Nova cellular data modem [38] and verified that the same data files we produced and transferred over WiFi in the field could be successfully sent to the same server via HTTP POST requests over Hologram’s cellular data network. Repeated tests in the laboratory showed that transfer of a single day of data (a 550 KB raw data file and an 8 KB disaggregated event data file) required approximately 0.25 s over WiFi versus 25.31 s over cellular. Cellular transmission is slower and would likely consume more power because the Raspberry Pi computer would be powered for longer; however, the cost of transmitting data over cellular is based on data size and not transmission time.

Emplacing the computational node’s weatherproof enclosure above ground made it physically exposed to be opened, compromised, or stolen. While the Raspberry Pi computer has basic endpoint security measures, such as a username and password required for login, it currently lacks data encryption both on the device and during data transfer, which may make it vulnerable to unauthorized access. This is likely not a strong concern because water use data are not highly sensitive. Even so, edge computing reduces the amount of data that are at risk at any one time because each computational node contains data for only a single home. A breach in the network would expose data from one node, whereas a breach in a centralized system may expose much more data.

Another potential limitation of the node is related to data importance. While we used the computational node to transfer both unprocessed and processed data to the server to demonstrate its capabilities, in the quest to minimize data transfer bandwidth and reduce latency, it is more practical to only transfer processed data that contains useful, decision-relevant information for water providers and users. In the event that the raw, unprocessed data are never transported to a centralized system and may never be saved on the computational node, important information that may be present in that data could be overlooked and discarded. There may be useful applications of the raw data (beyond end use disaggregation) that are unrealized because the raw data are not transmitted and stored long term. This is the specific reason why our design enables local saving and transmission of both raw and processed data. Local saving of raw data can be turned off to save space on the Raspberry Pi computer’s Micro SD card. Transmission of raw data can be turned off to minimize communications bandwidth and required centralized storage. Both can be temporarily turned on to enable monitoring and diagnostics of performance. Localized processing of data on the node may even reduce the amount of data to be transmitted to something that may be feasible over networks with much lower bandwidth than WiFi or cellular (e.g., LoRaWAN) [39], although we did not explore this option.

## 5. Discussion and Conclusions

The work presented here builds upon existing smart water metering and end use disaggregation studies to develop and demonstrate new, open source, and reproducible data collection, end use disaggregation, and classification methods that can be executed on existing water meters using relatively inexpensive hardware. In the context of our case study application, the record of classified events produced by the computational node is the same as what would be produced by logging data in the field followed by manual downloading and centralized post-processing of the high-resolution data. The type of data products that can be produced by the computational node have already been shown to support a wide range of analysis and modeling applications typically undertaken by interdisciplinary research and integrated water management teams.

The hardware designs and the firmware code used to prototype the computational node are available and open source. The device we designed can either be built using off-the-shelf components or it can be manufactured by a PCB manufacturer, providing flexibility for potential users who may not have electronics prototyping expertise. The computational node was successfully deployed and tested in a laboratory setting under optimal conditions (e.g., constant temperature with a dedicated power supply) and in the field under variable temperature and power configurations to demonstrate successful sensing, data logging, and computational capabilities on existing analog water meters. This means that for approximately $199.47, the computational node can be viably used with existing, magnetically-driven residential water meters to: (1) collect data at a very high temporal frequency and up to the pulse volume resolution of the meter, (2) extract and classify water end use events or other computational tasks directly on the node, and (3) transfer the unprocessed raw data and/or the classified water end use events to a centralized server without affecting the performance of the existing meter.

We anticipate that the device that we have prototyped could be used by researchers in data collection and processing to address questions about residential water use and user behavior, by water managers to collect and analyze data from residential settings for operational use, by homeowners to monitor their water use, and by water meter manufacturers to upgrade the designs of existing smart water meters. We believe that the hardware design of the computational node is generalizable across these potential users and their use cases, but this may require additional software to present the event data produced in a context understandable by the user. For example, researchers and water managers could likely parse and analyze event data using general purpose data visualization and analysis software, but homeowners may need additional levels of data aggregation or summary and a custom user interface (e.g., a smartphone app) designed to present the event data in an easily interpretable way.

While our case study application focused on end use disaggregation and classification, the computational capabilities of the node are generic. Similarly, the potential uses of the data are not constrained and could certainly include optimization programs. Any analysis or computational code required by the user can be executed by the Raspberry Pi computer. Processing data at the edge of the network close to where data are generated instead of centrally enables delivery of intelligent, near real-time responsiveness, while drastically reducing the amount of data that must be transferred. Because computational tasks such as end-use disaggregation are performed directly at the meter site, the results can be transferred to other devices with less (or almost no) computing power. This minimizes the required visits to sites for retrieving data and reduces the amount of processing power required to provide local (e.g., an in-home display or smartphone app) or centralized (e.g., aggregated data for a water utility) data services because the useful information has already been computed by the time it gets to the system on which it is used. Because computing is done before results are sent, water utilities do not have to wait (or pay) for data to transfer over a network or for it to be computed centrally and sent back, thus minimizing potential network latency. It also minimizes the need for central storage and management of large volumes of raw data. Although many services related to water use data do not need to be done in real-time, applications such as leak detection could be delivered in near real time to water providers and consumers. The current tradeoff for these capabilities is that the device’s owner must install and maintain the device, including its battery.

The results of computations (e.g., daily water use summaries and disaggregated end uses) are much smaller than the raw, high-resolution data and can be much more easily communicated over a network with a much lower bandwidth. This would be considerably cheaper on a cellular data network and more scalable on radio networks such as LoRaWAN, which can be relatively inexpensive but has lower available bandwidth for data transfer. In our case study application, one data collection site collected approximately 550 KB of data per day of raw data at the four second recording interval. This would be about 201 MB per year per site. In a small city with 5000 residential connections, this would add up to about 1 TB of data sent over a telemetry network per year if every meter were equipped with high-resolution data collection. The disaggregated event data files averaged less than 8 KB per day for the same site, which means that savings of multiple orders of magnitude in data size could be realized if only processed events are transmitted.

The computational node we designed for collecting, processing, and transferring high temporal resolution data advances available smart water metering and supporting cyberinfrastructure for building the scientific data and knowledge base for sustainably managing urban water supplies. We anticipate that our design and the concepts that we have demonstrated will be useful in building and managing next-generation smart metering systems and their resultant data.

The classified water end use events can equip water utilities and water users with a detailed information on the variation in water use for each end use type, including the total number of events, number of events per day, number of events per capita per day, event volume, event duration, and event flowrate for each of the end uses.

## Figures and Tables

**Table 1:** EEPROM data format.

Number of Bytes	Record Type
0–2	Number of data records
3–8	Starting timestamp
9-N	Data bytes

**Table 2:** Parts required and cost to build a CIWS computational node using off-the-shelf components.

Part	Cost ($)	Vendor
12 V 10 Ah Duracell Battery	39.99	Batteries + Bulbs
Raspberry Pi 3B Computer	35.00	Adafruit
Pelican 1150 Waterproof Case (with foam)	31.96	Amazon
3.3 V, 8 MHz Arduino Pro (ATmega328p Board)	15.95	Sparkfun
Datalogging Shield	15.95	Adafruit
Micro SD Card with Adapter	9.95	Mouser
5-Conductor Cable	9.11	Mouser
TSR_1-2450 Converter	5.48	Digikey
LIS3MDL Magnetometer + Breakout Board	4.95	Pololu
1725656 Terminal Block	1.66	Digikey
1725685 Terminal Block	4.10	Digikey
25LC1024-E/SM Connectors	3.09	Digikey
Anderson Powerpole Connectors	2.60	Amazon
Battery Lead Connectors	2.75	Grainger
Box Kit	2.10	Mouser
1920-1076-ND Cable Glands	1.75	Digikey
FQP27P06 MOSFET	1.78	Digikey
Strap Set: Gear Strapz (+5 Clasps)	1.52	Amazon
100 NF 50 V 0805 Capacitor	1.50	Mouser
1 µF Ceramic Capacitors	0.92	Mouser
Stripboard	1.43	Mouser
10 k Ohm Resistor	0.20	Digikey
4.7 k Ohm Resistor	0.10	Digikey
In-Line Fues Holder	4.21	Mouser
2N3904 Transistor	0.44	Mouser
Spacers	0.20	Mouser
Fuse	0.17	Mouser
Button	0.16	Mouser
Screws	0.17	Mouser
Nuts	0.12	Mouser
Serial Extender Housing Pack	0.16	Pololu
Total Cost	199.47	

**Table 3:** Functions executed by the CIWS computational node Computational_Firmware.ino file and the main objective.

Function	Main Objective
setup()	Executes the following tasks: Initializes the system state data structure. Initializes GPIO pins. Initializes the magnetometer sensor. Initializes the real-time clock. Initializes the AVR SPI module. Sets up the magnetometer and real-time clock interrupt handlers. Initializes Raspberry Pi computer report data. Stops using the clock for all unused peripherals to reduce power consumption.
loop()	The datalogger firmware’s main loop function that performs the following actions: Check if the Raspberry Pi computer activation button is pressed. Copy report data with the Raspberry Pi computer. Negotiate the SPI bus with the Raspberry Pi computer. Check if a data recording interval has elapsed. Update timestamp. Check if magnetometer data are ready. Process incoming data to count peaks.
INT0_ISR()	Checks if there are any new data ready to report.
INT1_ISR()	Checks if the data recording interval has elapsed.

**Table 4:** System State library functions.

Type	Function	Output
Byte	pulseCount()	The number of pulses in the current sample period.
Byte	lastCount()	The number of pulses in the previous sample period.
Byte	interval()	The time interval between data records.
Integer	totalCount()	The number of pulses since logging started.
Long	recordNum()	The record number of the current sample period.
Long	romAddr()	Pointer to the current address in EEPROM.
Bool flag	logging()	True if the device is logging, false if it is not.
Bool flag	flag4()	True if a data recording interval has passed, false if it has not.
Bool flag	readMag()	True if magnetometer data are ready, false if it is not.
Bool flag	newReport()	True if a transaction with the Raspberry Pi computer is complete, false if it is not.
Bool flag	RPiON()	True if power is supplied to Raspberry Pi computer, false if it is not.
Bool flag	powerGood()	True if the Raspberry Pi computer signals after power-on, false if it does not.
Bool flag	romFree()	True if the Raspberry Pi computer that signals it is finished with EEPROM, false if it is not.
Bool flag	RPiFalseON()	True if the Raspberry Pi computer is unresponsive on power-on, false if it is not.

**Table 5:** RTC library functions.

Function	Objective
rtcTransfer()	Responsible for transferring raw data collected on the microcontroller to the RTC and takes an eight-bit register number, a read/write flag, and an eight-bit value to write. This function utilizes the Arduino IDE’s Wire library for I2C communication with the RTC.
loadDateTime()	Reads all of the RTC’s date and time registers, and stores the resulting data in a date/time structure. This function is called each time a data recording interval has passed.
copyDateTime()	Reads data in a date/time structure and stores the data in a second date/time structure. This function is called when the Raspberry Pi computer is activated. The copied timestamp is the start time for the next batch of data in the EEPROM.
setClockPeriod()	Adjusts the RTC interrupt clock period, thus adjusting the time interval between data records.

**Table 6:** Communication library functions.

Function	Objective
updateReport()	Used to update system information and configuration based on data in a report from the Raspberry Pi computer. These reports are passed between the Raspberry Pi computer and the microcontroller one byte at a time.
powerRPiON()	Used to power on the Raspberry Pi computer by setting pin PC2 (microcontroller analog pin 2) high. This action triggers the power switching circuit, which connects the battery to a 5-volt regulator, which then powers the Raspberry Pi computer.
powerRPiOFF()	Used to power off the Raspberry Pi computer by setting pin PC2 low. This action turns off the power switching circuit, essentially disconnecting the Raspberry Pi computer’s regulator from the battery.
UART_Init()	Initializes the microcontroller’s UART module at a baud rate of 9600 bps (bits per second). The UART is only used to communicate with the Raspberry Pi computer.
UART_Transmit()	Takes an input byte and writes it to the UART data register, UDR0. The microcontroller automatically takes the data in UDR0 and transmits it on the UART Tx pin.
UART_Receive()	Reads the UART data register. Reading the data register loads the byte received on the UART Rx pin.
UART_End()	Disables the UART module.
spiInit()	Initializes the microcontroller’s SPI module, which is used for writing data to the EEPROM chip. The module is reactivated whenever a transaction is about to take place.
spiOff()	Deactivates the microcontroller’s SPI module.
spiSelectSlave()	Sets the SPI chip select pin low, signaling to the EEPROM that an SPI transaction is about to take place. This function also activates the SPI module.
spiReleaseSlave()	Sets the SPI chip select pin high, signaling to the EEPROM that the transaction has been completed. This function also deactivates the SPI module.
spiTranceive()	Iterates over the array of input bytes and writes each byte to the SPI data register (SPDR). The function waits while each byte is transmitted, then reads the SPDR.

**Table 7:** Results from laboratory experiment 1 for the 1-inch and 5/8-inch Master Meters.

Time		1-Inch Meter Water Use Volumes (L)			5/8-Inch Meter Water Use Volumes (L)		
	Flowrate (LPM)	Meter	Computational Node	Error (%)	Meter	Computational Node	Error (%)
9:00	0	0.00	0.00	0.00	0.00	0.00	0.00
9:10	17.6	191.36	191.36	0.00	191.36	192.54	0.62
9:20	30.8	289.12	289.44	0.44	288.60	290.28	0.58
9:30	54.4	518.4	519.04	0.49	517.16	519.80	0.51
9:40	71.2	756.8	759.39	1.35	757.35	762.31	0.66

**Table 8:** Results from laboratory experiment 2 for the 1-inch and 5/8-inch Master Meters.

Time		1-Inch Meter Water Use Volumes (L)			5/8-Inch Meter Water Use Volumes (L)		
	Flowrate (LPM)	Meter	Computational Node	Error (%)	Meter	Computational Node	Error (%)
9:50	0	0.00	0.00	0.00	0.00	0.00	0.00
10:00	13.80	147.20	147.20	0.00	147.20	147.24	0.03
10:10	35.2	351.20	351.52	0.09	348.87	350.34	0.42
10:20	55.2	472.64	473.28	0.14	469.74	477.25	1.60
10:30	64.8	618.08	625.12	1.13	621.60	633.24	1.87

## Data Availability

All of the hardware modifications, parts, PCB design, firmware code, and supplemental materials required for producing the computational node described in this paper are available in the GitHub repository for the project at https://github.com/UCHIC/CIWS-WM-Node (accessed on 15 June 2021). The repository contains separate folders for Hardware and Firmware. All of the firmware libraries (.h and .cpp files) and supplemental firmware documentation are available in the Firmware folder. The Hardware folder contains additional images of the logger, hardware design, layout, PCB design, and instructions to perform the hardware modifications described in this article. All of the data collected during the field deployment associated with the results reported in Section 4.3 and Section 4.4 are open source and publicly available in the HydroShare repository [40].
